# Using Rational Emotive Behavior Therapy to Improve Psychological Adaptation among Future Coaches in the Post-Pandemic Era

**DOI:** 10.3390/healthcare11060803

**Published:** 2023-03-09

**Authors:** Tomas Saulius, Romualdas Malinauskas

**Affiliations:** Department of Physical and Social Education, Lithuanian Sports University, LT-44221 Kaunas, Lithuania

**Keywords:** rational emotive behavior therapy (REBT), ABC(DE) framework, cognitive emotion regulation, university students, post-pandemic era

## Abstract

This study aims to demonstrate that applying rational emotional behavior theory (REBT) concepts can help to improve the psychological adaptation of future coaches in the post-pandemic era. The current research utilizes a qualitative approach with a sample of 14 future coaches, namely, students of the study program “Training systems” at Lithuanian Sports University. These students are also active athletes (basketball players). Interview questions were developed based on the ABC(DE) model, and answers were analyzed following the methodology of content analysis. In general, our findings fit the ABC(DE) framework. However, there are several important exceptions. During discussions, participants presented their basic beliefs in the form of statements rather than demands. The ABC(DE) framework predicts the latter. Furthermore, participants’ automatic thoughts are not logical derivatives of their basic beliefs, contrary to the REBT theory. However, it is possible to interpret basic beliefs as an explanation for automatic thoughts, meaning that this interpretation seemingly does not contradict the theoretical principles of REBT. As such, REBT appears to be helpful for better understanding the psychological adaptation problems that arose during the pandemic and are relevant in the post-pandemic era.

## 1. Introduction

As a matter of trivial fact, sport is a highly emotional activity; both competing athletes and their coaches experience an array of emotions during different phases of athletic performance (i.e., in the pre-competition, competition, and post-competition phases). Various negative emotions have demonstrated considerably larger effects during both the COVID-19 pandemic and the post-pandemic era, effects not only limited to elite athletes, but also impacting student athletes [1]. As such, it is safe to assume that the education of future coaches (who are usually actively performing athletes during their studies of sports coaching) must prepare them to manage emotions, whether the emotions are experienced by themselves or expressed in different ways by others, including by the athletes they train. In our paper’s introductory section, we first describe the emotional dimension of sports in more detail. Next, we introduce concepts that are essential for informing the emotional intelligence of future coaches, with the main aim of linking the notions of emotional regulation and the ABC(DE) framework, which includes five stages: Activating event, Beliefs, Consequences, Disputation, and Effective new beliefs. Rational Emotive Behavior Therapy (REBT) is based on that framework. By exploring these concepts, the paper introduces the central research question: how can REBT help future coaches manage spontaneous negative emotions during the post-pandemic era?

In the post-pandemic era, the student population has faced psychological problems, demonstrating “a larger effect of the pandemic situation in their psychological variables, presenting higher levels of anxiety (state and trait), depression, acceptation/resignation coping style, higher neuroticism (emotional instability trait), and lower agreeableness trait” [2] (p. 1). Most of the studies analyzed by one systematic review [3] reported negative psychological effects in the post-pandemic era, leading the researchers to conclude that “in the post-pandemic era, college students have some new psychological characteristics: depression and anxiety, awakening and confusion, fragmented learning, networked socialization, and some of them have negative emotions and psychological problems such as panic, anger, and rebellion” [4] (p. 800).

Notably, the reduction of training during periods of strict quarantine demanded by the COVID-19 pandemic “has been shown to have [had] negative effects on athletic/sports performance” [5,6] (p. 1). In the post-pandemic era “anxiety, anger, and longing were the most commonly experienced emotions among athletes” [7] (p. 79), and anxiety can be interpreted as “worry regarding future competitions and sports performance” [7] (p. 79).

Regarding the significance of emotions in sport, there are a few points of importance [8,9,10]. First, unpleasant (negative) emotions do not necessarily have negative consequences for athletic performance, and pleasant (positive) emotions do not necessarily have positive consequences. Second, each athlete names their own emotions very specifically, and verbal labels for individual emotions tend to be idiosyncratic, with 80–85 percent of athletes’ self-identifying emotions not included in standardized questionnaires [8]. Third, trying to perform at their best (to demonstrate their mastery), different athletes experience emotions that differ in content and intensity. That is, each athlete has an individualized emotion profile [8]. For example, let us consider the individualized emotion profiles of two athletes [8]. The first athlete’s optimal emotions are both negative (“vehement”, “attacking”) and positive (“motivated”, “charged”, “brisk”, “resolute”, “active”). The second athlete achieved a high level of performance feeling different optimal emotions. Meanwhile, the first athlete’s response to their performance was both negative (“irritated”, “dissatisfied”, “tense”) and positive (“motivated”, “purposeful”, “willing”, “excited”). It is evident from Yuri Hanin’s interviews with athletes that emotions become optimal (conducive to goal achievement) by virtue of not of being pleasant (positive) or unpleasant (negative) but of how an athlete succeeds in subordinating them to their actual needs. To illustrate this, consider the following: “When I feel angry, it usually has a negative influence on me. I start finding faults with others and that feeling is so strong that I can’t handle it (I mean on ice). And I get stupid penalties and often fight with the opponent. But sometimes, I use my angry feelings when I need more aggressiveness in my game” [8] (p. 12). Thus, the state of mind of an athlete during their performance is far from apathetic and unemotional, and different emotions, regardless of their content, if handled appropriately, can produce a successful performance. In both sports and other activities, we aim to eliminate dysfunctional (i.e., unhelpful, “undesirable”) emotions and induce functional (helpful, “desirable”) emotions. This is the essence of emotion regulation [11,12,13,14]. According to one definition, “emotion regulation refers to the strategies people use to influence their own emotional experience” [13] (p. 596), with the results of James J. Gross’ research indicating that 9 out of 10 people apply one or another emotion regulation strategy at least once a day [11].

So-called “cognitive models of emotion”, which constitute scientific alternatives to the “common sense model” of folk psychology, assume that events cause emotional and other reactions not directly but by mediating thoughts, beliefs, evaluations, imaginations, and memories [12,15]. In other words, emotional reactions differ according to stimulus interpretation (the way individuals make stimuli meaningful to them). Notably, the cognitive model developed by the psychotherapist Albert Ellis [16] is employed by this study due to its significant practical importance for the education of future coaches.

According to Jordana, Turner, Ramis, and Torregrossa [17], although only one journal article (and one doctoral thesis) on the application of REBT appeared during the first decade of the new millennium, publication on the topic accelerated during the second decade, with 2020 seeing 15 journal publications (39 total scientific studies when including doctoral theses, congress materials, etc.). Nonetheless, this scientific research avenue remains a path largely untrodden, even if it seems quite promising. It is clear that REBT appears to be a relevant approach, especially when one confronts the very important issue of the well-being (including mental) of athletes [18].

**Figure 1:**
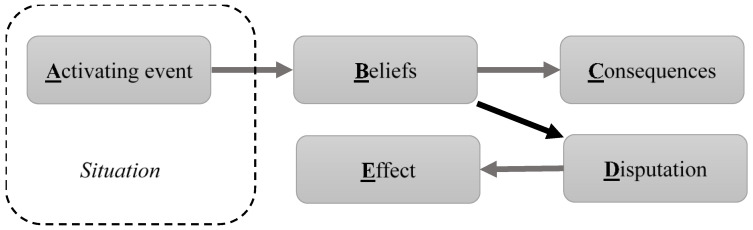
The main components of the ABC(DE) model (adapted from Waltman and Sokol [15]).

During the early stages of REBT’s development, the aforementioned framework comprised only three elements and was known as “the ABC model”. Over the subsequent decades, Ellis’ cognitive model has been elaborated, with the addition of more components, ultimately leading to the ABC(DE) mode (Figure 1).

The components of the ABC(DE) model can be summarized as follows [16,19]:

- Situation. Our emotional disturbances appear in specific situations. That is, psychological drama always unfolds in particular settings, whether in actual or imagined contexts or in the present, past, or future.
- Activating event or Adversity (A). A more-or-less obvious aspect of a situation provokes an emotional reaction, namely, something that upsets us in the particular situation. It can be a real event (such as an injury in sports) or an imaginary event (e.g., a player’s reading of what their coach meant by saying, “I’m proud of you!”). It can be a past, present, or future event. Finally, it can be an internal event (e.g., a headache) or an external event (e.g., the aggressive behavior of a teammate or an opponent).
- Beliefs (B). This is a system of irrational beliefs that is considered the real cause of emotional crisis. It is a person’s “personal philosophy” or “core philosophy of life” that is dysfunctional. Irrational beliefs can be conscious or unconscious. Furthermore, as mentioned earlier, such beliefs can be fundamental or derivative (inferential). From the perspective of REBT, our dysfunctional (irrational) belief system comprises (a) our “absolutistic demands”, which form the core of this system, and (b) “irrational derivatives of these demands”, which include so-called “awfulizing”, “low frustration tolerance”, and “global evaluations of worth” [20] (p. 41). REBT helps to identify and discard fundamental beliefs (“demands”) that dictate a distorted, unrealistic interpretation of a particular event and lead to psychologically undesirable consequences.
- Consequences (C). These are the negative effects of irrational beliefs on various aspects of personality. Consequences can be cognitive, can appear as negative “automatic thoughts” (e.g., “Their team is much stronger than ours”, “I am a loser”), distractions of attention, impaired memory, etc. Consequences can also be emotional (e.g., pre-performance anxiety, distress, or fear during a performance), physiological (e.g., muscle tension, tremors, or increased sweating), or behavioral (e.g., attempting a three-point shot from a disadvantageous position).
- Disputation (D). This is a process of psychotherapeutic intervention that involves identifying and challenging our irrational beliefs. As mentioned, irrational beliefs can be rejected empirically (i.e., by demonstrating that they do not correspond to facts or to reality), logically (i.e., by demonstrating that different beliefs a person holds to be true actually contradict each other), or pragmatically (i.e., by demonstrating that beliefs do not lead to the desired results). Furthermore, beliefs can be disputed from the perspective of flexibility: if a person assumes a dogmatic position or presents themselves as holding a monopoly on truth, it can be shown to them that such an epistemic stance prevents them from acquiring new knowledge and new competencies, thus significantly diminishing their personal progress.
- Effective new beliefs (E). These are rational beliefs that, via patient–therapist interchange (disputation), replace old irrational beliefs. Such changes in the “philosophy of life” allow for better adaptation and promote psychological well-being. In the best-case scenario, basic irrational beliefs, placed under the heading of “demands” (or “shoulds, oughts, and musts”), are replaced by so-called “preferences”, “awfulizing” is replaced by “anti-awfulizing”, “low frustration tolerance” is replaced by “high frustration tolerance”, and, finally, “global evaluations of worth” (or “damnations”) are replaced by “unconditional acceptance”.

Among the main assumptions of REBT is the idea that negative emotions are not necessarily dysfunctional and are to be avoided at all costs [20]. Our beliefs largely determine the health of negative emotions, with rational beliefs underpinning healthy negative emotions (HNEs) and irrational beliefs underpinning unhealthy negative emotions (UNEs) [19]. According to theoreticians and practicians of REBT, the following equations hold true: A × B = eC (Activating event × Belief = Emotional Consequences; reads as “An Activating event multiplied by a Belief equals Emotional Consequences”, where the arithmetical symbol of multiplication captures the strong impact of our belief on our interpretation of a particular situation and, consequently, our emotional life) [19]. Thus, we have the following: A × iB = UNE (Activating event × Irrational Belief = Unhealthy Negative Emotion) and A × rB = HNE (Activating event × Rational Belief = Healthy Negative Emotion). For example, in the situation where the activating event is a threat, irrational beliefs produce anxiety (UNE) and rational beliefs promote concern (HNE) [17]. Thus, identifying, questioning, and discarding the irrational beliefs deeply entrenched in our worldview is essential for effectively applying the REBT method.

Thus, REBT exemplifies the emotional regulation techniques that Gross’ [11] classification labels “cognitive change strategies”. Based on the previous discussion of the theoretical principles of REBT, three relevant points can be made: First, emotional reactions correlate with the contents of inner talk that are propositional (and, usually, normative) in nature. Second, thoughts that constitute our inner talk are inferences (derivatives) of fundamental beliefs that usually evade introspection (our conscious reflection) and become evident only under more elaborate scrutiny (e.g., when the ABC(DE) framework is applied). Third, modification of fundamental beliefs conforms to the rational criteria of flexibility, logical consistency, truth, and utility, affecting both inner talk and emotions. The REBT technique has been tested in different settings, with a sample of university students showing that REBT represents an effective method for coping with anxiety [21]. Studies have also confirmed its effectiveness for athletes of different ages [22,23]. The current study aims to reveal whether and how the use of REBT concepts can help improve the psychological adaptation of future coaches in the post-pandemic era. To do so, the paper investigates the extent to which the qualitative data collected conform to the ABC(DE) framework, the model upon which REBT is based.

## 2. Materials and Methods

The current research has adopted a qualitative approach for several reasons. First, it enables the investigation of human experience and behavior in immediate real-world contexts. Second, it is sensitive to the specifics and dynamics of the subject matter under consideration. Third, it enables, over the course of the preliminary (pilot) research, the determination of different subject-matter variables and the tentative identification of those with the most promising explanatory value, in doing so providing empirical grounds to form working hypotheses for future quantitative research [24]. Among the array of approaches to qualitative research, content analysis was chosen as the main method of our research due to its focus on phenomena that cannot be observed directly and that enable the development of an explanatory account of the phenomena under consideration with an eye on “factors, impacts, [and] influences” [25] (p. 47). In particular, content analysis usually involves the following components: (a) purposeful, or theoretical, (instead of random) sampling according to the analytical needs of the research; (b) word-by-word, line-by-line (etc.) initial data coding (or conceptualizing); (c) gradual development of codes into categories and categories into themes; (d) memoing, which refers to making informal notes about emerging categories and themes, about comparisons of the data at hand, and about perspectives on and directions for further data collection; (e) constant comparison of, for example, new data with already constructed categories, new data with old data, and already constructed categories with new categories; (f) saturation, that is, ceasing to collect information if the acquisition of new data becomes irrelevant to emerging codes, categories, or themes [26].

Content analysis was chosen for this study to identify whether and to what extent empirical data confirms the hypothesis that critical reflection and correction of fundamental beliefs contribute to better (i.e., more functional, adaptive) emotional states among future coaches, as one would assume on the basis of the main assumptions of REBT). In other words, our research represents an attempt to identify the theoretical pattern that emerges from empirical (qualitative) data and compare this pattern with that presupposed by REBT theory.

Our research sample included 14 second- and third-year undergraduate students from the “Training systems” study program at Lithuanian Sports University (seven men and seven women). All participants play on the university’s (men’s and women’s) basketball teams. Purposive sampling was applied because it is recommended for qualitative research in general [27].

The research included two main phases. In the first (introductory) phase, all participants were given a 90-min group lecture on the essence of emotion regulation and REBT as a version of cognitive change strategy. In the second phase, participants were interviewed individually (face-to-face). Questions from the researcher and answers from participants were recorded and then transcribed verbatim. Individual interviews took 138 min on average (usually with breaks of 10–15 min). Both phases of the research were conducted at Lithuanian Sports University in January 2022 and February 2022 (i.e., during the post-pandemic period). Before each individual interview, participants were given brief information regarding the aims of the research and guarantees regarding anonymity and the confidentiality of the data collected during the research. Informed consent was obtained from all individual study participants.

Semi-structured interviews were adopted as the data collection method. We prepared a series of questions corresponding to the ABC(DE) framework:

- Situation: Can you remember any situation in your recent sporting experience in which you felt negative emotions? I am asking about any emotionally stressful situation during a match, before a match, or after a match.
- Activating event (A): What exactly made you feel poorly in the situation you have just mentioned? Pick one thing as the main cause of your emotions in that situation.
- Consequences (C): What exactly did you feel in the situation we are talking about? What did you say to yourself in that situation? What thoughts captured your attention while having these feelings (emotions)?
- Beliefs (B): So, you had these feelings (emotions) and these thoughts in the situation we are discussing. Maybe another person would not have the same feelings and thoughts in the same situation, would they? Let us suppose, just suppose, that it is something you firmly believe that made you feel and think the way you felt and thought in the situation we are talking about. What might it be?
- Disputation (D): So, we have decided about a thing (let us call it “belief”) that makes you feel bad and have bad thoughts. Let us suppose that this thing (belief) has nothing to do with reality. Let us suppose it is a false belief. If so, there must be strong reasons against it. What could these reasons (arguments) be?
- Effect (E): So, we have decided that there are reasons (arguments) against believing that … [content of the belief]. How could you benefit from discarding this belief? How could it affect, in a positive way, the thoughts and/or feelings that occur in situations resembling the situation we were talking about at the beginning of our conversation?

During the research, these prepared questions were complemented by additional questions usually intended to clarify the meaning of the answers given by participants. Thus, these questions were generally the “What do you mean by saying …?” and “Could you explain (or elaborate) …?” type. In some cases (e.g., talking about consequences), additional questions were asked to elucidate the most essential aspects of respondents’ answers (e.g., “Among these various thoughts, which was the most prominent?”). Regarding the series of questions prepared for our interviews, two points should be made clear. First, the component “Beliefs (B)” and the component “Consequences (C)” switch places in the series above to facilitate the identification of basic beliefs (one could reasonably assume that identifying causes is easier when one first explicitly defines consequences). Second, to make our interviews as short as possible, questions about consequences have been exclusively related to emotional and cognitive consequences, leaving physiological and behavioral consequences aside.

## 3. Results

This section of the paper presents the results of this research’s qualitative study. In the main text of the section and in the tables accompanying the text, the seven male participants are coded M1 to M7, and the seven female participants are coded F1 to F7. As mentioned, interviews with the participants were recorded and transcribed, with analysis of this material involving coding, categorizing, and thematizing according to the precepts of the content analysis method. Notably, because the series of questions prepared for the semi-structured interview was based on a theoretical model—namely, the ABC(DE) framework—this theoretical predisposition of the research is naturally reflected in the interpretation (analysis) of the results. Because the series comprises six question phases, the coding and categorizing of the answers predictably produced six main themes in the further analysis. In other words, our study applied what is commonly called “theoretical coding” (also known as “selective coding” or “conceptual coding”), something that is not unusual in grounded theory research [28]. According to Thornberg and Charmaz [29], “theoretical codes consist of ideas and perspectives that researchers import to the research process as analytic tools and lenses from outside, from a range of theories. Theoretical codes refer to underlying logics that could be found in pre-existing theories” [29] (p. 159).

The first theme, “Situation”, comprises three categories. As Table 1 shows, participants usually associated “emotionally stressful situation” with their performance during a match or, more precisely, with the crucial final moments of a match, when winning or losing strongly depended on their performance (code “Trying to score in the final minutes of a match”). This is fully predictable from the fact that all participants are basketball players in the student basketball league. The following is the most representative answer of this type:

You see that time is ticking and you understand: “Now or never”. They [opponents] know what you are going to do. Of course, they’ll attempt to make a foul. And you perfectly know how to move to score. In a moment, it appears that everything is in your hands, but suddenly everything goes astray […] You go astray.

(M1)

Results for the second theme, “Activating event” (Table 2), are more dispersed. Four codes appear somewhat more frequently than other codes: “Calling names”, “Rough play of an opponent”, “Failure to play according to the coach’s instructions”, and “Expectations of others”.

Notably, for participants, an activating event was typically one or another element of the external reality, that is, the social environment: it was other people who engaged in verbal abuse (F2, F4) and aggressive play (M5, M6), gave instructions that participants failed to follow (M1, M3), or placed the burden of their expectations on participants (F1, F7). However, there is one important exception where the activating event was more internal than external, that is, more associated with psychological reactions than social stimuli:

Usually, I feel weird after a match. It is difficult to describe […] I feel tired, I say “Good night” to everybody and go to bed earlier. Maybe, I just want to be alone. But I don’t fall asleep […] When I am alone, I am thinking about what happened and about what could happen. I understand that I could perform better […] Of course, others expect something from me, because I am a team player. I expect something from them [teammates] and they expect from me […] Not that they say or require something from me, but I myself can understand it.

(F7)

The theme “Consequences” (Table 3) features two main categories: “Negative emotions” and “Negative self-talk”. These categories were likely pre-determined by the established intention to focus interviewing on precisely these aspects.

In the category of “Negative emotions”, the code “Feeling angry or furious” evidently appears most frequently. In the category “Negative self-talk”, the “Negative evaluations of other persons” code is most common. Considering the principles of REBT, it is notable that negative evaluations of other persons (F2, F4, M5, M6, M7) correlate with external stimuli, including the overt physical (M5, M6) and verbal (F2, F4, M7) behavior of these persons and with such emotional reactions as feeling angry (F2, M5, M6) and feeling hurt (F4, F7). However, the assumption of causation cannot be justified here because comparison of the results in Table 3 also reveals that sometimes (M1) an individual’s anger also corresponds with negative evaluations of their own personality. Of course, these considerations are quite consistent with the theoretical assumption of REBT that negative emotions are associated with negative evaluations as such, regardless of the object of these evaluations [16]. Finally, the finding that “Negative evaluations of other persons” is most frequent in the category “Negative self-talk” is quite consistent with the previous finding that participants tended to identify activating events––that is, triggers of their emotional reactions––among individuals that belong to their social environment.

The following exemplifies responses coded “Negative evaluations of other persons”:

I thought then, “You f*****g asshole! Watch your elbows! I also can play like this!” Maybe I said this to him, not only thought it. I don’t remember […] When afterward I was in a locker room, I talked to others [teammates] and thought that such assholes should [not] be allowed to play basketball.

(M5)

As Table 4 shows, the theme “Beliefs” includes three types of beliefs: beliefs about the world, beliefs about other people, and beliefs about oneself. However, these beliefs rarely manifested in an imperative form (demand), with the exception of the code “I should be more professional”. Instead, participants usually presented these in the form of statements (or “factual claims”). Participants identified demands to themselves (e.g., “I should be more professional” type) as underlying their emotional reactions in several cases: feeling “numb” during a warm-up drill and becoming anxious and fearful of the consequences (F3); making a technical mistake at a decisive moment in a match, feeling angry about themselves, and criticizing their own actions (F5); failing to score because of the psychological pressure and feeling upset and becoming critical of their actions (F6); engaging in post-performance ruminations, feeling “squeezed” and devaluating their personality (F7); failing to play according to the coach’s instructions and becoming upset and critical about their personality (M3). In these cases, it is hard to be certain whether the imperative to be “more professional” reflects the respondent’s conscious beliefs about their actions or their personality. It could be both. Because most participants who gave answers of this type (F3, F5, F6, F7) play on the same female basketball team, it might be safe to assume that this imperative reflects what participants constantly hear from their coach. That is, in these cases, we encounter something that could be termed “internalized beliefs of significant others”.

As mentioned, most participant beliefs were expressed as statements (not imperatives), with “I am too emotional as a person” representing the most frequent code. Of course, this statement logically implies the imposition, “I should not be as emotional a person as I am”. However, none of the participants made this explicit. Meanwhile, it is notable that participants tended to evaluate themselves as overemotional persons in quite different situations: not only when they did something or failed to do something (F1, M1, M3) but also when they were exposed to the wrongdoings of other people (F2, F4, M6).

The interviews exposed a quite definite logic underlying these self-evaluations: regardless of what may happen, of who other people are and what they do, a person should blame themselves for their emotional overreactions to these things. Because the participants are athletes who have undergone extensive physical and psychological training, such logic may reflect their practical and theoretical knowledge about psychological toughness. Among those participants who evaluated themselves as overemotional persons, several also held the belief that “Sport is all about winning” (F1, M1). However, the scarcity of such cases prevents any generalizations being made. The same must be said about cases in which the same participants held the belief “I am too emotional as a person” and one or another belief about other people, such as that “People are cruel” (F2, F4) or that “Some athletes have no idea how to compete appropriately” (M6).

The following interview excerpt exemplifies responses coded “I am too emotional as a person”:

Every time we fight for a position, she [opponent] speaks nonsense and calls me different names. I say to her: “F**k off!” But she keeps doing it. Maybe seeing that I weigh more, she tries to use it to her advantage. I mean psychologically. Because she can do nothing physically […] I mean that people are cruel and try to use as a weapon something that other people are uncomfortable with […] It makes me very angry, to the extent that I can suffer […] I am too emotional as a person. I know this very well.

(F2)

In general, the fact that participants tend to identify as their core belief some variation of the notion that “I am too emotional as a person” reflects the difficulties that participants had interpreting the content of their negative self-talk as the logical consequence of their underlying beliefs. For example, in the case of participant M1, there is no direct connection between the automatic thought “What a fool I am!” and the core belief “Usually, emotions engulf me too easy”. In this case, as in others (F2, F4, M6), it would be quite incorrect to say that automatic thoughts follow logically from core beliefs (as REBT assumes).

Let us proceed to the classification of arguments intended to discard dysfunctional or irrational beliefs (see Table 5). Notably, this classification includes only those arguments that participants themselves had accepted as true and relevant in the face of their beliefs. Arguments based on values (Olympic values or universal human values) were of the rarest type, with only two cases belonging to this category (M5, M6). Their use and effectiveness were limited because, defending their beliefs, participants were quick to take a position of so-called “ethical relativism” (e.g., “so what that they [other people] think this way? I have a different opinion”, F2). Participants were more receptive to arguments from utility, especially those that emphasized mental health (F2, F5, F6, M4). As an example, let us consider the case of a participant who identified being physically exhausted during the final minutes of a match as the most emotional situation in his recent experience (M4). He described feeling upset in this situation and tending to see his lamentable physical state among the reasons for his team’s failure. He also declared such beliefs as “Sport is all about winning” (about the world) and “I am not the one who loses with an easy heart” (about himself). In the discussion phase, when challenged about his reasons for engaging in activities that he acknowledged liking and that have nothing to do with winning (or some other external result) and are pleasant by themselves, he admitted that these activities are conducive to a “good psychological state”. Finally, he was unable to deny that playing basketball could be an activity of the very same type. However, this illustration also makes it apparent that arguments on the basis of mental health were not introduced separately, with their effectiveness depending on interconnection with arguments of another type, namely, those coded as “Noting inconsistency”. 

Interestingly, Table 5 makes it evident that “Noting inconsistency” is the most frequent code in the theme “Discussion” (F1, F3, M1, M2, M3, M4). It is notable that appeal to logical inconsistency represents an effective tool for managing various beliefs. This includes not only beliefs about oneself––for example, “I am too emotional as a person” (F1, M1, M3), “I should be more professional” (F3), and “I am not the one who loses with an easy heart” (M4)––but also beliefs about the world, such as “Sport is all about winning” (F1, M1, M4) and “Life is unfair” (M2). One could hypothesize that the argumentative efficacy of “Noting inconsistency” reflects a tendency to eliminate “cognitive dissonance” [20]. However, the interconnectedness of critical arguments requires withholding conclusions regarding which type of argument is the most effective. 

Table 6 shows the main categories and the codes that belong to the theme “Effect”. Recall that, according to REBT theory and our research design, “Effect” is an umbrella term for significant changes in the belief systems and emotional lives of participants.

Regarding changes in participants’ belief systems, REBT theory suggests that new (rational, functional) beliefs oppose or contradict older (irrational, dysfunctional beliefs). For example, if one holds the belief, “I absolutely must not be rejected”, a counter belief might be, “I’d prefer not to be rejected, but it doesn’t mean that I must not be”. Changes in the emotional life of participants are more complicated, because there is no consensus regarding how notions of “unhealthy emotions” and “healthy emotions” should be defined. Some researchers (e.g., Ellis) are inclined to consider these different types of emotions. However, other researchers (e.g., Dryden) claim that these notions describe differences in intensity rather than type [19].

At any rate, consideration of “healthy” and “unhealthy” emotions are largely irrelevant to our research: Participants tended to be very unspecific about changes in their emotional life, with eight of the 14 participants (F2, F4, F5, F6, F7, M1, M2, M3) defining this desirable change in terms of “Becoming emotionally resilient” (see Table 6). Emotional resilience (also rendered “emotional toughness”, “emotional stability”, “psychological toughness”, “psychological balance”) was emphasized by participants with beliefs about themselves including “I am too emotional as a person” (F2, F4, M1, M3) and “I should be more professional” (F5, F6, F7, M3), beliefs about other persons including “People are cruel” (F2, F4), and beliefs about the world including “Sport is all about winning” (F6, M1) and “Life is unfair” (M2). Of the six participants who mentioned considering themselves overly emotional (F1, F2, F4, M1, M3, M6), four (F2, F4, M1, M3) described desirable change in terms of emotional resilience. However, none were able to explain the notion of emotional resilience in an informative way. A circular definition was usually given (F1, F2, M1, M6), such as “Emotional toughness is when you resist your emotions […] ‘Resisting emotions’ means that you are psychologically strong. It is when you are stronger than your emotions” (F2). Some participants claimed that this notion is self-explanatory (F2, F4, M3, M6), and some acknowledged being confused about the meaning of the notion (F1, M1).

Regarding changes in belief systems, the codes “In the course of life, values of things, such as victory, change” (F1, F6, M1, M4) and “Overcoming self-doubt” (F3, F7, M3, M6), from the categories “Changing beliefs about the word” and “Changing beliefs of oneself”, are the most frequent. As mentioned, REBT assumes that a change in belief system involves not merely replacing one belief with another (regardless of the contents) but instead concerns the logical procedure of advancing antitheses of previous beliefs. In general, our study’s results conform to this scenario. In some cases (F1, F6, M1, M4), participants intend to change their old belief “Sport is all about winning” to the new belief, “In the course of life, values of things, such as victory, change”. Here, the new belief is the evident antithesis of the old belief. The same can be said about cases (F3, F7, M3) in which participants initially believed that they lacked professionalism (“I should be more professional”) and intended to develop a more positive self-conception (“Overcoming self-doubt”). In such cases, it is safe to conclude that it was easier for participants to see logical alternatives to their fundamental beliefs than to infer automatic thoughts from basic beliefs (as noted in the discussion of Table 4).

## 4. Discussion

In recent years and especially during the post-pandemic period, the psychological adaptation problems of student athletes has received limited scholarly attention. Accordingly, this study has been undertaken to explore the extent to which REBT can help students adapt psychologically in the post-pandemic era.

Recall that our study identified anger as the most common emotional reaction in stressful situations, especially in-game situations, as reflected by the codes “Conflict with an opponent” and “Trying to score in the final minutes of a match” (see Table 3). According to Tuncel [30], “anger is an emotional response to a suffered injury or injustice” [30] (p. 74). Common triggers of anger (in the sporting context) include the following: intentional or unintentional fouls by opponents, mistakes by teammates, misjudgments of referees, fan attitude, poor performance, and bad field conditions [30]. It is evident that most of these anger triggers pertain to an athlete’s social environment. Social influence on an individual’s emotional life can be both overt (in the form of verbal and nonverbal messages) and covert (in the form of inferences regarding what other people want, think, or mean). The research by Turner et al. vividly illustrates the latter. For example, an athlete can perceive their bad performance as a problem because of the expectations or evaluations of the coach (e.g., “The coach might think I’m not good enough for the academy”) [31] (p. 80) or family members (e.g., “[…] there’s people you know, they want you to win”) [32] (p. 296). Finally, it is important to note that in team sports, emotional reactions do not necessarily represent an individual’s perception of a situation, with group identity frequently a factor [30].

Our second finding is that social factors become triggers of negative reactions under the condition that these factors are interpreted in a particular way, that is, they receive negative evaluations. This finding echoes the main principle of REBT: “[W]hat people feel and do stems largely from the way they actively and creatively construct and reconstruct reality rather than from their passively reacting to it” [33] (p. 299). Our study’s findings reveal that although participants tend to perceive other persons in a negative light, it is more common to encounter negative perceptions of oneself, whether of one’s personality, one’s actions, or the consequences of one’s actions. This is consistent with Ellis’ claims that “ideas and feelings about self-worth” are at the core of emotional problems because they are “largely definitional and are not empirically confirmable or falsifiable” [16]. That is, making claims about who we are and how well we fare, we usually rely on selective autobiographical memories and treat personality as an unchanging substance rather than the sum of different processes. It is a widely accepted position that self-awareness is particularly important in sports because it represents a precondition for self-esteem and motivation [14]. However, there exists substantial evidence that we are susceptible to “introspection illusion”, that is, inclined to develop inadequate, mistaken conceptions of our values, motives, and personal traits [34].

Let us discuss our third finding, namely, that participants’ basic beliefs are rarely identified by participants as “demands” (imperatives). As mentioned, REBT builds on the assumption that so-called “demands” (or “absolutistic demands”) exist at the core of emotional disturbances. In general, these demands are of three types: I must perform well, always succeed, and gain the approval and admiration of other people; people must always be fair to me, accept my opinions, and follow my example; the world must be comfortable, unchallenging, and generous to me [16]. For Ellis, these demands constitute the “irrational philosophy” of a person, that is, a general interpretative scheme according to which one makes one’s experiences meaningful [16]. In this respect, Ellis follows famous psychoanalyst Karen Horney, who writes that despite “should” (or “inner dictates”) exerting “enormous coercive power” on a person, they are usually “too difficult and too rigid” [35] (p. 72, p. 85). This suggests that there is a strong psychoanalytic flavor in the theoretical basis of REBT, despite various declarations of hostility towards psychoanalysis made by Ellis and his followers. Regarding the application of REBT in sports contexts, researchers usually feel quite confident in identifying the irrational demands of participants. For example, in the case of elite-level archers suffering from strong pre-performance and performance anxiety, researchers have identified the following underlying demand: “I would like to perform well when I compete in relatively easy competitions, therefore I must, if not it would be awful, and this would be unbearable for me” [36] (p. 81). In the case of a 12-year-old tennis player troubled about his performance (i.e., making unforced errors), researchers acknowledged a certain “resistance” on the part of the participant to associating his emotional overreactions with a demand. Nonetheless, they concluded that his irrational belief could be justifiably identified as the following demand: “Professionals don’t make unforced errors; therefore, I must not” [37] (p. 45). Not pressing the issue any further, it seems sufficient to note that there is an important difference between a belief reported and a belief interpreted, and researchers must maintain constant awareness of this distinction.

Our study’s fourth finding concerns a logical gap between automatic thoughts (the propositional contents of so-called “inner-talk”) and the beliefs supposedly underlying them. That is, these two are not connected (at least directly) as conclusion and premise. It can be said that the belief “Usually, emotions engulf me too easy” underlies the automatic thought “What a fool I am!”; however, the notion “underlies” carries the rather general meaning of “being relevant to understanding this situation and other similar situations”. In this case, a basic belief can function as a sort of explanation for disturbing and repetitious automatic thoughts. Analogically, basic beliefs about oneself, such as “I am too emotional as a person” can be said to shed meaning on or explain the automatic thought “I am too emotional as a person”. However, this possibility is not reflected in the theory and practice of REBT, even if it is consistent with REBT. It is possible that problems with the logical status of underlying beliefs—as encountered in our study—concern their status as unconscious thoughts (as assumed by REBT). For example, Ellis claims that the beliefs underlying our emotional problems can be both conscious and unconscious [16]. He assumes that there are “the unconscious or unaware ideologies that lead us to behave neurotically” while also noting that such belief systems, or ideologies, “are usually by no means as deeply or mysteriously hidden as the classical psychoanalysts stubbornly still believe” [33] (p. 174). Of course, remembering that the practice of REBT is a time-consuming process [18], each participant in our study was interviewed for only two hours on average, with the logical gap between automatic thoughts and basic beliefs reported by participants interpretable as reflecting our study’s limitations. However, it should be noted that the very idea of “thinking of one’s thinking” can be quite alien to athlete participants [18]. Thus, it is a dubious endeavor to press such participants to give responses that fit the ABC(DE) model but that only superficially capture their beliefs or even contradict those beliefs.

Our fifth finding is that “noting inconsistency” appears to be quite a universal tool in critical discussions and in subsequently eliminating dysfunctional beliefs. According to Ichino [38], superstitious beliefs and confabulations share many common characteristics, including a tendency to overlook empirical evidence. Consequently, they are usually contradictory and internally and externally inconsistent (with other knowledge and beliefs). One can hypostasize that this property can be attributed to unrealistic beliefs, including those that are of great importance to REBT. For example, for Ellis [10], thinking, which tends to become absolutistic (too demanding), inevitably generates inconsistent beliefs; in other words, “unrealistic notions, when rigidly held, frequently contradict each other and thwart the individual’s realistic goals” [16] (p. 51). This means that it is possible to challenge these notions and demonstrate their inconsistency via various technical means (e.g., by introducing facts or analogies). Sometimes, REBT practitioners introduce their method as “Socratic questioning” [36].

Finally, it is apparent that participants demonstrate more logical competence in finding alternatives to their dysfunctional beliefs than in identifying those beliefs as the premises for their automatic thoughts. As demonstrated earlier, among those participants who identified the idea “I am too emotional as a person” as their basic belief, most ultimately agreed (after discussion) that this personality trait is not something given or fixed in its nature and identified as a desirable solution (effect) “Becoming emotionally resilient”. On the one hand, this preference manifests their readiness to change and better adapt to their social environment, which represents the major goal of REBT [16]. On the other hand, as in the case of basic beliefs, desired effects are stated as claims rather than imperatives, with further interpretation needed to make them the “preferences” that REBT seeks. More significant deflection from the REBT model is evident in cases where participants identified the following beliefs as desirable effects: “In the course of life, values of things, such as victory, change” and “If someone hurts you, it does mean that everybody is bad”. It is possible that these results reflect our study’s limitations.

The present research’s results agree with data produced by a previous qualitative study [39] that explored the symptoms of students’ psychological stressors and their causes and provided essential remedies for coping with such behaviors in the post-pandemic era, revealing that “unusual behavior, lack of confidence, improper sleep, and lack of motivation were identified as the primary symptoms of students’ stress” (p. 240).

Strengths, limitations, and future prospects.

The strengths of the present study include its use of REBT in a cross-cultural setting. Research of this nature is useful for further assessing the validity of REBT conceptualizations and principles. The findings provide additional confirmation of the utility of REBT principles. 

Concerning the limitations of the current research, a convenience sample comprising 14 respondents is too small to reflect the characteristics of the population under study. As such, far-reaching generalizations should be avoided. Future research should explore the use of REBT concepts with larger and different samples in other locations. That is, while the findings of this case study are promising, they need further assessment in other settings. To assess the effectiveness of REBT over time, further research should be conducted, preferably longitudinal.

## 5. Conclusions

Based on our empirical results, we can make several conclusions. First, in various situations (pre-performance, performance, post-performance), social factors (namely, other people) are the major triggers of negative automatic thoughts and emotions. Second, one or another aspect of the social environment (for instance, the post-pandemic era) becomes a trigger of negative automatic thoughts and emotions when a person tends to interpret it as something unacceptable (i.e., negatively evaluate this aspect). Third, according to participants, beliefs underly their negative reactions but very rarely assume the form of “demands” or imperatives (at least, for participants themselves). Fourth, for participants, an “underlying belief” (or “basic belief”) is not necessarily a belief from which automatic thoughts follow logically. Fifth, appealing to logical inconsistency is a quite universal critical tool for addressing dysfunctional beliefs (even if its effectiveness depends on applying arguments of other types). Finally, participants find logical alternatives to their dysfunctional beliefs that are derived logically from considering their automatic thoughts in relation to those underlying dysfunctional beliefs.

Generally speaking, during discussions, participants not only identified their main emotional issues but also envisaged sensible ways of resolving those issues. In this respect, REBT––as a set of tools for critical reflection, can help to improve the psychological adaptation of future coaches in the post-pandemic era.

## Figures and Tables

**Table 1:** Theme “Situation” with its categories and codes (F—abbreviation for female participants, M—abbreviation for male participants).

Theme	Categories	Codes	Distribution of Codes among Participants
Situation	Pre-performance	Trying to rest before a match	F1, M2
		Warm-up drill before a match	F3
	Performance	Conflict with an opponent	F2, M5, M6
		First quarter of a match	M3
		Trying to score in the final minutes of a match	F5, F6, M1, M4
	Post-performance	Chatting with team-mates in a locker-room	F4
		An eye-to-eye conversation with coach	M7
		Being alone after a match	F7

**Table 2:** The theme “Activating event” with its categories and codes.

Theme	Categories	Codes	Distribution of Codes among Participants
Activating event	Physical issues	Painful injury/minor trauma	M2
		Feeling numb (“paralyzed”)	F3
		Physically exhausted	M4
	Actions of another person	Calling names	F2, F4
		Unjust remarks of a coach	M7
		Rough play of an opponent	M5, M6
	Technical failures	Failure to play according to the coach’s instructions	M1, M3
		“Foolish fault”	F5
	Psychological pressure	Importance of a match for a player	F6
		Expectations of others	F1, F7

**Table 3:** The theme “Consequences” with its categories and codes.

Theme	Categories	Codes	Distribution of Codes among Participants
Consequences	Negative emotions	Feeling angry or furious	F2, F5, M1, M5, M6
		Feeling upset	F6, M3, M4
		Feeling hurt	F4, M7
		Feeling “as a squeezed lemon”	F7
		Feeling anxious	F1, F3, M2
	Negative self-talk	Negative evaluations of one’s actions	F5, F6
		Negative evaluations of the consequences one’s actions	F3, M2, M4
		Negative evaluations of one’s personality	F1, F7, M1, M3
		Negative evaluations of other persons	F2, F4, M5, M6, M7

**Table 4:** The theme “Beliefs” with its categories and codes.

Theme	Categories	Codes	Distribution of Codes among Participants
Beliefs	Beliefs about the world	“Sport is all about winning”	F1, F6, F7, M1, M4
		“Life is unfair”	M2
	Beliefs about other persons	People are cruel	F2, F4, M5
		Some athletes have no idea how to compete appropriately	M5, M6
	Beliefs about oneself	“I should be more professional”	F3, F5, F6, F7, M3
		“I am too emotional, as a person”	F1, F2, F4, M1, M3, M6
		“I am not the one who loses with an easy heart”	F5, M4

**Table 5:** The theme “Discussion” with its categories and codes.

Theme	Categories	Codes	Distribution of Codes among Participants
Discussion	Arguments from utility	“Something is a right thing if it is good for your mental health”	F2, F5, F6, M4
		Emphasizing better social relationship	F4, F7, M5
		Putting emphasis on performance, not victory	F1, F5, F7,
	Arguments based on values	Olympic values	M5
		Universal moral principles	M6
	Argument based on logical standards	Noting inconsistency	F1, F3, M1, M2, M3, M4
		Noting hasty generalizations	F2, F4, F6

**Table 6:** The theme “Effect” with its categories and codes.

Theme	Categories	Codes	Distribution of Codes among Participants
Effect	Changing beliefs about the world	Challenges are inevitable	F2, M5
		In the course of life, values of things, such as victory, change	F1, F6, M1, M4
	Changing beliefs of oneself	Overcoming overconfidence	M2
		Overcoming self-doubt	F3, F7, M3, M6
		Placing emphasis on one’s actions, not one’s personality.	F1
	Changing emotions	Becoming happy	F5, M4
		Becoming emotionally resilient	F2, F4, F5, F6, F7, M1, M2, M3
	Changing beliefs about other persons	“It is important to communicate with people, not to judge them”	M5
		“If someone hurts you, it does not mean that everybody is bad”	F4

## Data Availability

Please contact authors for data requests.
